# Retraction of “Eriodictyol corrects functional recovery and myelin loss in SCI rats”

**DOI:** 10.1515/tnsci-2022-0275

**Published:** 2023-02-14

**Authors:** Chenggang Li, Chunfang Wang

**Affiliations:** Department of Orthopaedics, Second Hospital Shanxi Medical University, Taiyuan, Shanxi, 030001, China; Laboratory Animal Center, Shanxi Medical University, Taiyuan, Shanxi, 030001, China

The article “Eriodictyol corrects functional recovery and myelin loss in SCI rats” by Chenggang Li, Chunfang Wang (https://doi.org/10.1515/tnsci-2020-0128) has been retracted as per the authors’ request.

The authors found that the results shown in this study are not reproducible and should not be used for conclusions.

